# MeInfoText: associated gene methylation and cancer information from text mining

**DOI:** 10.1186/1471-2105-9-22

**Published:** 2008-01-14

**Authors:** Yu-Ching Fang, Hsuan-Cheng Huang, Hsueh-Fen Juan

**Affiliations:** 1 Institute of Molecular and Cellular Biology, National Taiwan University, Taipei 106, Taiwan; 2 Institute of Biomedical Informatics, National Yang-Ming University, Taipei 112, Taiwan; 3 Department of Life Science, National Taiwan University, Taipei 106, Taiwan; 4 Graduate Institute of Biomedical Electronics and Bioinformatics, National Taiwan University, Taipei 106, Taiwan; 5 Center for Systems Biology and Bioinformatics, National Taiwan University, Taipei 106, Taiwan

## Abstract

**Background:**

DNA methylation is an important epigenetic modification of the genome. Abnormal DNA methylation may result in silencing of tumor suppressor genes and is common in a variety of human cancer cells. As more epigenetics research is published electronically, it is desirable to extract relevant information from biological literature. To facilitate epigenetics research, we have developed a database called MeInfoText to provide gene methylation information from text mining.

**Description:**

MeInfoText presents comprehensive association information about gene methylation and cancer, the profile of gene methylation among human cancer types and the gene methylation profile of a specific cancer type, based on association mining from large amounts of literature. In addition, MeInfoText offers integrated protein-protein interaction and biological pathway information collected from the Internet. MeInfoText also provides pathway cluster information regarding to a set of genes which may contribute the development of cancer due to aberrant methylation. The extracted evidence with highlighted keywords and the gene names identified from each methylation-related abstract is also retrieved. The database is now available at .

**Conclusion:**

MeInfoText is a unique database that provides comprehensive gene methylation and cancer association information. It will complement existing DNA methylation information and will be useful in epigenetics research and the prevention of cancer.

## Background

DNA methylation, occurring predominantly in CpG dinucleotides, is an important epigenetic modification of the genome that is involved in mediating various cellular processes [1]. DNA methylation has a wide range of biological functions, including an essential developmental role in the reprogramming of germ cells and early embryos, the genomic imprinting, the X chromosome inactivation, the repression of endogenous retrotransposons and the generalized role in gene expression [2]. Abnormal methylation of DNA may result in increased transcription of oncogenes or silencing of tumor suppressor genes and is common in a variety of human cancer cells [3]. Although the ramifications of global hypomethylation for tumor development are less well understood, it might contribute to chromosomal instability and then increases in gene expression [4,5]. The hypermethylation of CpG islands in gene promoter regions is associated with aberrant silencing of transcription and has been regarded as a common mechanism for inactivation of tumor suppressor genes in human cancer [3,6]. As compared with normal cells, the malignant cells show major disruptions in their DNA methylation patterns [7] and some genes seem to be aberrantly methylated in a tumor-specific manner [2]. Currently, many studies have corroborated the DNA methylation profile of a cell type which may serve as a biomarker with a diagnostic and prognostic value [8]. In addition, the initiation of the process of abnormal promoter methylation may associate with chromatin-remodelling complexes [5,9]. Therefore, if the contribution of each candidate gene to tumorigenesis can be proved, the exact methylation profiles of tumors are available and the molecular events that initiate and maintain epigenetic gene silencing are understood clearly, then the prevention and treatment of cancer could have come more focused and rational [5,10].

Text mining in biology is to automatically extract specific information about genes, proteins and their functional associations from text documents [11]. As more biological literature is published electronically, it is desirable to develop methods for automatic extraction of relevant information from any source of biology data, especially from sources such as literature written in human language (also known as natural language) [11,12]. And the development of text-mining applications specific for biology is the only way to cope with the increasing amount of free textual data produced in this field [11]. Over the past few years, a considerable number of studies have been made on the mining and extraction of information from biomedical literature, such as disease candidate genes [13,14], protein-protein interactions [15,16], protein functions [17] and modifications [18]. Many different approaches such as named-entity recognition (NER) based on a dictionary, template or ontology; statistics of word co-occurrence and natural language processing (NLP) have been adopted or invented by various researchers to achieve the goal. However, so far no attempt has been made to analyze the available DNA methylation information from a vast amount of literature.

In this paper, we present a biological database providing gene, methylation and cancer association information mined from the text and integrated protein-protein interaction and biological pathway information. Since MethDB [19] is the only public database that was developed to store information containing the origin of the investigated sample, including experimental procedure and DNA methylation data, it could be anticipated that our database is able to act complementarily to the existing databases, fill a gap in the already available DNA methylation resources and facilitate the research on epigenetics.

## Construction and content

### Data sources and contents

**Figure 1:**
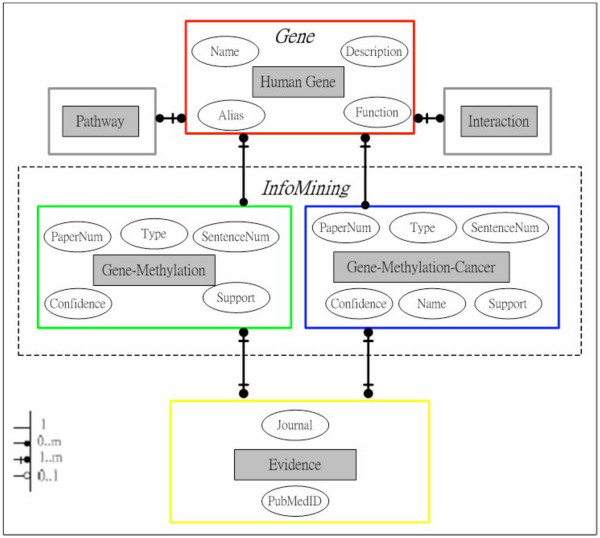
**The simplified relational scheme of MeInfoText**. Each gray box represents an entity with various major attributes characterized by oval-shape. For instance, each human gene may contribute to one or more cancers due to abnormal methylation, have many interacting partners and involve several signaling pathways. Each association between gene methylation and cancer could be referred to one or more evidences.

MeInfoText is a relational database implemented by MySQL and Perl programming language in the Linux environment. Figure 1 shows the simplified relational scheme of our database. For example, each human gene in our database may associate with one or many cancers due to abnormal gene methylation, such as hypermethylation. Each association could be referred to more than one known evidences extracted from the biomedical literature.

**Figure 2:**
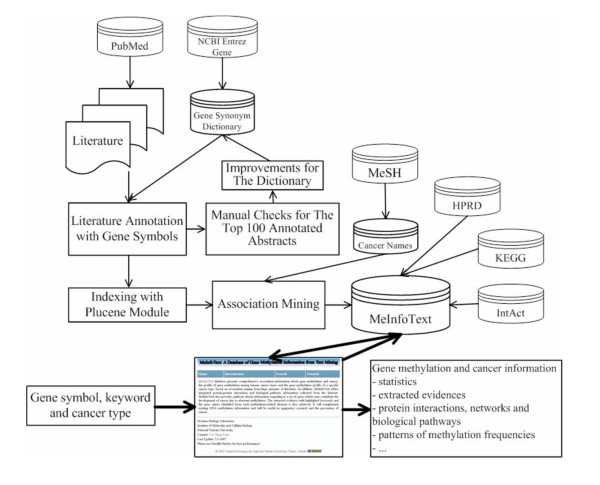
**The text mining approach and information integration for MeInfoText**. Literature about human, methylation and cancer is collected from PubMed and annotated with gene symbols. The most recent 100 gene-annotated abstracts are manually checked to reduce false named entity recognitions and enhance dictionary coverage. The gene-annotated documents are indexed with Plucene module and then mined according to the frequencies of co-occurrences of entities. Various association, protein-protein interaction and pathway information are stored in the relational database, MeInfoText. Users can search the database via the web interface. Thick arrow indicates the basic workflow of MeInfoText.

MeInfoText contains associations among human genes, methylation and cancers and integrated information about protein-protein interactions and biological pathways. The general human gene information, including official gene symbol, aliases, description and function was retrieved from NCBI Entrez Gene [20]. At present, 17425 human genes are available in our database. The protein-protein interaction data was collected from HPRD [21] and IntAct [22]. It provides information on interacting partners, interaction types and detection methods. The biological pathway information collected from HPRD and KEGG [23] describes pathway types, regulations for genes, and experiments. The gene methylation-related pathway cluster information is automatically generated using literature mining results and known pathway data. Cancer types were obtained from the medical subject headings vocabulary (MeSH). All association information was mined from MEDLINE abstracts collected through PubMed with query terms including human, methylation and cancer. Figure 2 shows our text mining approach and information integration for constructing MeInfoText.

### Gene synonym dictionary

We constructed a human gene synonym dictionary containing official gene symbols and aliases to annotate gene names in the literature. To make sure that most gene information stored in our dictionary is validated experimentally, we first collected all human protein entries from Swiss-Prot, a curated protein sequence database, and retrieved corresponding gene information, including official gene symbol, aliases, full name and summary from NCBI Entrez Gene. Information regarding to human miRNA genes was directly obtained from NCBI Entrez Gene. The annotation process was based on pattern matching between the dictionary entries and words in abstracts. The match was case-insensitive and only whole words were matched. After the complete of initial identification, we manually examined most recent 100 gene-annotated documents to reduce false named entity recognitions and enhance dictionary coverage. If unexpected words were frequently matched in the documents, these ambiguous gene synonyms would be regarded as stop words and removed from the dictionary. On the other hand, if no gene synonym was able to be found in the document by the dictionary, the document would be checked manually and then the discovered gene synonyms, if any, would be added to our dictionary. We then annotated gene names in the literature again with the improved dictionary.

### Cancer type vocabulary

Cancer types were obtained from the heading fields in the Neoplasms by Site (C04.588) section of the MeSH. If a cancer type is composed of tumor site and Neoplasms i.e. Breast Neoplasms, in order to increase vocabulary coverage only the site name would be added to our cancer name set for matching abstracts and sentences. Other cancer types whose suffixes are -oma such as retinoblastoma were directly added to the set. Neoplasm-related keywords including cancer, tumor, tumour, neoplasm and -oma were first used to check if abstracts or sentences might describe cancers. If any, the set of cancer names would be used to discover what kinds of cancers occur in abstracts or sentences. We then mined associations between gene methylation and cancer based on term co-occurrences. For example, "BRCA1 methylation contributes to a subset of sporadic cancers of the breast." could be first identified by neoplasm-related keyword, cancer, and then by the site name, breast. Furthermore, BRCA1, methylation and breast cancer could be found they occur together in a sentence.

### Information retrieval

The gene-annotated documents were indexed with Plucene module, a perl search engine toolkit based on the Lucene API [24], to allow fast access to words stored inside the text. The associations between genes, methylation and cancers were mined according to their co-occurrences in the full abstracts and sentences. The keywords for methylation include 'methyl-', 'hypermethyl-', 'hypomethyl-', 'histone' and the methods of detecting DNA methylation, such as 'MSP' and 'COBRA'.

### Association rules

We used confidence and support to measure our association rule interestingness [25]. Let *I *be a set of items. Let *D *be a set of database transactions where each transaction *T *is a set of items such that *T *⊆ *I*. An association rule is an implication of the form *A *→ *B*, where *A *⊂ *I*, *B *⊂ *I*, and *A *⋂ *B *= Φ. The rule *A *→ *B *holds in the transaction set *D *with support *s*, where *s *is the probability that item *A *and *B *occur together, *P*(*A *⋃ *B*). The rule *A *→ *B *has confidence *c *in the transaction set *D *if *c *is the conditional probability of *B *given *A*, *P*(*B*|*A*). In other words, confidence is the frequency of entries containing item *A *and *B *within all entries containing item *A *and support is the frequency of entries containing item *A *and *B *within all entries.

For a specific gene g, the set *I *consists of methylation terms, cancer terms, and gene g. A transaction *T *corresponds to a sentence within all the retrieved abstracts concerning the gene *g*. The association rule *A *→ *B *is defined by *A *= {gene *g*, methylation} and *B *= {cancer}, that is (gene *g*, methylaion → cancer). Hence we can calculate the support and calculation as follows:

$$\text{Support}={\left(\frac{\text{number of sentences with g, methylation, cancer }}{\text{total number of sentences}}\right)}_{\text{abstracts with g}}\;$$

$$\text{Confidence}={\left(\frac{\text{number of sentences with g, methylation, cancer }}{\text{number of sentences with g, methylation}}\right)}_{\text{ abstracts with g}}\;$$

Here, we use BRCA1 gene as an example. Currently, of all the abstracts mentioning BRCA1 gene in our database, there are a total of 1095 sentences. Among them, 167 sentences contain BRCA1, methylation and cancer; 251 sentences contain BRCA1 and methylation. Thus, support of the association rule (BRCA1 gene methylation → cancer) is (167/1095) * 100% = 15.3%; confidence is (167/251)*100% = 66.5%.

## Utility

### Query method

**Figure 3:**
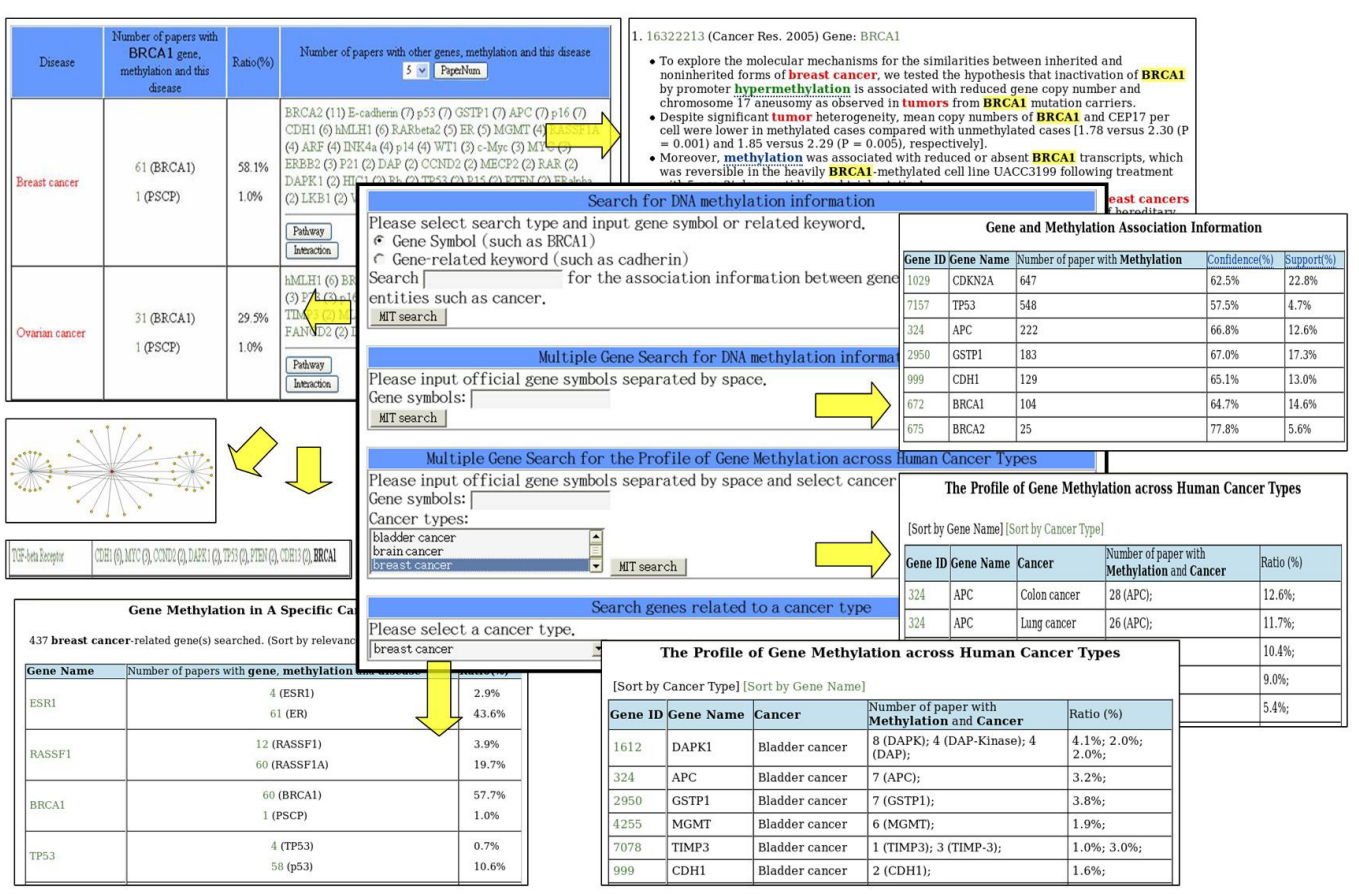
**The search interface and various features of MeInfoText**. The central box represents the MeInfoText search interface composed of four categories, (1) search for associations among gene, methylation and cancer, (2) multiple searches for gene methylation associations, (3) multiple searches for the profile of gene methylation across human cancer types and (4) search for gene methylation of a specific cancer type. The major search results are shown around. Literature evidences with highlighted keywords could also be retrieved.

MeInfoText can be accessed by gene symbols, gene-related keywords and cancer names to find information about genes, associations among genes, methylation and cancers, interacting partners, biological pathways, the profile of gene methylation across human cancer types, gene methylation profile of a specific cancer type, and extracted evidences from existing literature. In this study, the profiles of gene methylation mean the patterns of frequency of gene methylation that is primarily represented by the number of related literature. Figure 3 shows the search interface and various features of MeInfoText.

In order to retrieve gene methylation and cancer information regarding the human BRCA1 (breast cancer 1, early onset) gene, for example, users can specify 'Gene Symbol' search type, input BRCA1 as search term and then press the MIT search button. If the search is matched, the general gene information, including Gene ID, Swiss Prot accession number, Gene Name, Gene Aliases and Description, is shown. Users can then follow the NCBI Entrez Gene ID link for statistics and association information. The returned web page contains information about gene function, cross-references, association between BRCA1 gene methylation and cancer in sentence level, protein-protein interactions, biological pathways and statistics, including the numbers of papers and sentences containing gene methylation, hypermethylation, hypomethylation, histone and stem cell. Users can follow the link of "Does the gene encode a methyltransferase?" to tell if a gene encodes a methyltransferase by support evidences, if any, automatically extracted from Entrez Gene summary, GeneRIF, Gene Ontology [26] and Swiss-Prot [27]. For example, the parts of extracted evidences about HRMT1L2 (HMT1 hnRNP methyltransferase-like 2) are "protein methyltransferase activity [Gene Ontology]" and "Methylates SUPT5H. [Swiss-Prot] ". In the final part of the returned page, the information about co-occurrences of BRCA1 and different cancer types in the abstracts can be retrieved. Furthermore, one can see which other genes are also involved in a specific cancer type. Minimum number of papers of these genes is able to be specified. Default value is two. Users can then examine the pathway clusters and interactions of these genes. In the page of "Interaction Information about Gene Methylation and Cancer", the interaction network could be retrieved by following the link of Graph. Each node represents a gene, the red one represents the query gene such as BRCA1 and the blue ones represent other genes related to methylation. Each edge means an interaction between two genes. Specific association information and evidence extracted from the literature can be accessed by following the link of the number of papers containing gene, methylation and cancer. Information extracted from the literature is presented with highlighted keywords, identified genes, journal names and publication years.

To find gene methylation profile of human cancer, users may select a particular cancer type such as Breast cancer to find a set of genes undergoing abnormal methylation shared by this cancer. In addition, users can enter multiple official gene symbols separated by space to access gene methylation associations. For example, users are able to input 'BRCA1 APC CDKN2A GSTP1' to simultaneously retrieve association information including the number of related papers, confidences and supports. Users also can input multiple official gene symbols separated by space and select multiple cancer types to examine the profile of gene methylation across human cancer types. For instance, if a set of genes, including APC (adenomatous polyposis coli), BRCA1, CDH1 (cadherin 1), GSTP1 (glutathione S-transferase pi), MGMT (O6-methylguanine-DNA methyltransferase) and TIMP3 (TIMP metallopeptidase inhibitor 3), are inputted and different cancer types, including colon, lung, breast, gastric, liver, esophageal, bladder, leukemia, kidney, ovarian, head and neck, pancreas and lymphoma are selected, the profile of methylation for each gene among different cancers could be shown. Users may find the profile is cancer-specific or gene-specific.

### Inference of the pathways responsible for the development of tumors in a specific tissue

In addition to allow users to understand quickly the cancers in which a specific gene may play a role, MeInfoText could infer the pathways responsible for the development of cancers. If a set of genes could be associated to a given disease, they may tend to be connected at the translational levels. In other words, their gene products may interact with each other and involve in a biological pathway. For example, MeInfoText allows the user to query for APC gene and shows the cancers in which the gene may be methylated. Also, it shows the other genes that may be methylated for each of those cancers. In the 70 papers that mention APC gene methylation and colorectal cancer, there are 10 papers mentioning MLH1 (mutL homolog 1), 7 papers mentioning TP53 (tumor protein p53) and 3 papers mentioning MYC (v-myc myelocytomatosis viral oncogene homolog), all of which could be clustered into colorectal cancer pathway. Furthermore, TP53, CTNNB1 (catenin, beta 1), MYC and SFRP1 (secreted frizzled-related protein 1) are able to be clustered into Wnt signaling pathway and the interaction data indicates CTNNB1 has physical and direct interaction with APC. Therefore, users may infer abnormal methylation of these genes involve colorectal cancer-related mechanism and Wnt signaling pathway is responsible the development of tumors in colon tissue [28].

## Discussion

Our gene synonym dictionary, cancer names and mined associations were evaluated respectively. We used precision and recall measurements to estimate the performances Precision and recall are defined as follows: $\text{precision}=\frac{TP}{TP+FP}$; $\text{recall}=\frac{TP}{TP+NP}$ where *TP*, *FP *and *NP *are the number of true positives, false positives and negative positives. The data used for evaluating the gene synonym dictionary can be accessed at [29]. Partially identified gene symbols were considered false positives (i.e. "SKR-1 vs. SKR"). The precision and recall of our dictionary to recognize gene symbols in the test data are 96% and 74%. The data used for the cancer name evaluation can be retrieved at [30,31]. Non-specific terms such as cancer and tumor were excluded. There are about 382 text localizations tagged by <DIS></DIS> or <DISONLY></DISONLY> and mentioning cancer names. Partially identified cancer names were considered false positives (i.e. "B cell lymphoma vs. lymphoma" or "squamous cell lung cancer vs. lung cancer"). The precision and recall of cancer name recognitions are 79% and 67%.

We tested our association rules using human gene methylation and cancer data published by Das and Singal, 2004 [10] (reporting 13 genes commonly methylated in cancer, called dataset D) and Esteller, 2005 [3] (reporting 36 genes hypermethylated in cancer, called dataset E). In addition, human genes having methylation information and available in MethDB were also tested (dataset M, 18 genes). First, the ratios of relationships between gene methylation and cancer that could be discovered by MeInfoText are 13/13 (100%), 34/36 (94%) and 18/18 (100%) for datasets D, E, and M, respectively. Second, the average confidences and supports for the three test sets are (D: 61.3%, 17.1%) and (E: 60.6%, 17.5%) and (M: 54.4%, 16.2%). We observed that over 85% and 90% of the tested genes have confidences and supports greater than 40% and 7%, respectively. Therefore, we believed rules that satisfy a minimum confidence threshold, 40%, and a minimum support threshold, 7%, in this study are significant.

We evaluated the 75 associations in relation to the 34 unique genes and various specific cancers from Das and Singal, 2004 and Esteller, 2005. The data used for the evaluation are available at [32]. Mined associations with no explicit evidences that can support it were regarded as false positives. The precision and recall of association mining are 99% and 93%, respectively. Furthermore, we randomly selected 20 genes, PYCARD, CDH13, COX2, DAPK1, ESR1, GATA4, SYK, MLH1, TP73, PRDM2, PGR, SFRP1, SOCS1, SOCS3, STK11, TMEFF2, THBS1, RASSF5, PRKCDBP and RARB, from the 34 genes and manually evaluated the mined associations with at least 2, 3 and 5 papers, respectively. The number of the associations is 362, 222 and 103 and precisions are 78%, 85% and 91%, respectively.

MeInfoText might provide methylation markers for the detection of human cancer. From the MeInfoText search, we can find gene methylation profile of almost every human cancer type. The most relevant methylation-associated silencing of genes for each cancer could be combined into a set of potential markers which may reach high cancer detection information. For example, there are 342 genes whose abnormal methylation might relate to lung cancer and the top 11 genes having at least 20 papers include CDKN2A (cyclin-dependent kinase inhibitor 2A), RASSF1 (Ras association domain family 1), TP53, MGMT, DAPK1 (death-associated protein kinase 1), RARB (retinoic acid receptor, beta), HRAS (v-Ha-ras Harvey rat sarcoma viral oncogene homolog), RB1 (retinoblastoma 1), APC, FHIT (fragile histidine triad gene) and GSTP1. The different compositions of 3 or 4 of these potential markers may reach different cancer detection information and further investigation is required to support the usefulness.

Epigenetic inactivation may affect many existing cellular pathways [33]. For instance, through the MeInfoText search, we can find that the abnormal silencing of GSTP1 gene is strongly related to prostate cancer that may be also associated with aberrant methylation of other 20 genes with at least 3 papers. The pathway cluster information indicates both of GSTP1 and PTGS2 (prostaglandin-endoperoxide synthase 2) involve the TNF-alpha pathway, GSTP1, PTGS2 and TIMP3 participate in the IL-1 pathway and both of GSTP1 and CD44 (CD44 antigen) are components of the B cell receptor pathway. In addition, GSTP1 also involve glutathione metabolism and metabolism of xenobiotics by cytochrome p450. It is likely the methylation-associated silencing of multiple genes in different cellular pathways integrated by GSTP1 has a role in the progression of prostate tumors. In addition, the analysis of aberrant methylation of genes and inactivated pathways may help to understand the tumor behavior [3]. Although much more investigation is required to support the network of multiple genes and putative biomarkers for tumor progression, it would assist the understanding of mechanistic factors involving in such a process.

Epigenetics has been regarded as an important field to contribute cancer research and it is known that DNA methylation is not an isolated event but may be regulated by many complex epigenetic mechanisms such as histone modifications [34]. It has been suggested that loss of acetylation at Lys16 and trimethylation at Lys20 of histone H4 is a common hallmark of human cancer [34]. In addition, some studies suggest that DNA methylation may be related to the interactions between DNA methyltransferases, methyl-CpG binding proteins, histone deacetylases and histone methyltransferases and epigenetic information embodied in residue methylation states would flow from histone to DNA and back [35]. Therefore, we included "histone" as one of the keywords for methylation in our association mining.

### Comparison between MeInfoText and MethDB

The MeInfoText differs from the MethDB in many ways. Firstly, we have mined associations among genes, methylation and cancers from a large amount of biomedical literature. The computationally organized information would contribute epigenetics research. Secondly, MeInfoText provides the information about the profile of gene methylation among human cancer types and gene methylation profile of a particular cancer type. It would be useful to discover a set of potential markers for the detection of human cancer. Thirdly, MeInfoText provides integrated information about protein-protein interaction and biological pathway. Users can quickly overview which genes with aberrant methylation may contribute a cancer through various signaling pathways.

### Future developments

Future research might focus on database content and dictionary coverage increases. In addition, we would like to apply machine learning or other NLP techniques to do DNA methylation information extraction and compare the results with the study.

## Conclusion

MeInfoText is a novel database providing gene methylation and cancer association information from literature mining and integrated protein-protein interaction and pathway information. It facilitates researchers to comprehensively understand the relationships between multiple gene methylation and various cancers, the profile of gene methylation across human cancer types and gene methylation profile of a specific cancer, and to infer putative signaling pathways involving the development of tumors. It will complement existing DNA methylation information and be valuable to epigenetics research and the prevention of cancer.

## Availability and requirements

Project name: MeInfoText

Project home page: 

Operating system(s): platform independent

Other requirements: Firefox is recommended for the website access

License: the database website is freely accessible

## Authors' contributions

YCF carried out the database development, design, programming, data collection, text mining analysis, web interface and drafted the manuscript. HCH helped with the text mining analysis. HCH and JHF provided constructive suggestions for improving the website and helped to draft the manuscript. All authors read and approved the final manuscript.
